# Characterization of Muscle Fatigue Degree in Cyclical Movements Based on the High-Frequency Components of sEMG

**DOI:** 10.3390/biomimetics10050291

**Published:** 2025-05-06

**Authors:** Kexiang Li, Ye Sun, Jiayi Li, Hui Li, Jianhua Zhang, Li Wang

**Affiliations:** 1 College of Mechanical and Material Engineering, North China University of Technology, Beijing 100144, China; likex_2023@ncut.edu.cn (K.L.); 2024312070127@mail.ncut.edu.cn (Y.S.); 2 College of Mechanical Engineering, Hebei University of Technology, Tianjin 300401, China; 3 College of Mechanical Engineering, Beijing University of Science and Technology, Beijing 100083, China; 4 College of Electrical and Control Engineering, North China University of Technology, Beijing 100144, China

**Keywords:** human–robot interaction systems, muscle fatigue, sEMG, median frequency, EEMD

## Abstract

Prolonged and high-intensity human–robot interaction can cause muscle fatigue. This fatigue leads to changes in both the time domain and frequency domain of the surface electromyography (sEMG) signals, which are closely related to human body movements. Consequently, these changes affect the accuracy and stability of using sEMG signals to recognize human body movements. Although numerous studies have confirmed that the median frequency of sEMG signals decreases as the degree of muscle fatigue increases—and this has been used for classifying fatigue and non-fatigue states— there is still a lack of quantitative characterization of the degree of muscle fatigue. Therefore, this paper proposes a method for quantitatively characterizing the degree of muscle fatigue during periodic exercise, based on the high-frequency components obtained through ensemble empirical mode decomposition (EEMD). Firstly, the sEMG signals of the estimated individuals are subjected to EEMD to obtain the high-frequency components, and the short-time Fourier transform is used to calculate the median frequency (MF) of these high-frequency components. Secondly, the obtained median frequencies are linearly fitted, and based on this, a standardized median frequency distribution range (SMFDR) of sEMG signals under muscle fatigue is established. Finally, a muscle fatigue estimator is proposed to achieve the quantification of the degree of muscle fatigue based on the SMFDR. Experimental validation across five subjects demonstrated that this method effectively quantifies cyclical muscle fatigue, with results revealing the methodology exhibits superiority in identifying multiple fatigue states during cyclical movements under consistent loading conditions.

## 1. Introduction

The estimation of muscle fatigue based on sEMG signals holds significant value in multiple dimensions within the field of bionics. In the research and development of bionic robots, it serves as a fundamental basis for optimizing motion control strategies [1]. By analyzing muscle fatigue states reflected in sEMG signals, it enables dynamic adjustment of the motion parameters of robotic joints, such as torque and speed [2]. This not only enhances the motion efficiency but also reduces energy consumption, making the robot’s movements more natural and fluid [3]. In scenarios of human–robot interaction, it empowers the robot to perceive the operator’s muscle fatigue. Consequently, the robot can provide timely assistance, which greatly improves the comfort and safety of human–robot collaboration, allowing the bionic robot to better adapt to the operator’s physiological state and operational requirements [4]. In the realm of biomedical prosthetics, this technology has remarkably elevated the control precision of prosthetics. Bionic prostheses can precisely match the movement intentions of residual limb muscles and adapt to changes in muscle fatigue [5]. This not only enhances the wearing experience but also improves the usability of the prosthesis.

Human motion intention recognition technology has been extensively implemented in human–robot interaction systems, where sEMG signals serve as crucial biological information carriers for the robotic perception of human movements [6]. However, under complex human–robot collaborative scenarios, prolonged and intensive physical activities induce muscle fatigue that significantly alters muscular physiological characteristics, which is a critical factor affecting the accuracy and stability of human–robot interaction systems [7]. This phenomenon leads to progressive shifts in both time-domain and frequency-domain features of sEMG signals, consequently degrading the accuracy of motion intention identification [8,9,10]. Quantitative assessment of muscle fatigue levels during dynamic motions could effectively compensate for the adverse effects of physiological variations on action recognition precision in human–robot interactive processes.

**Figure 1:**
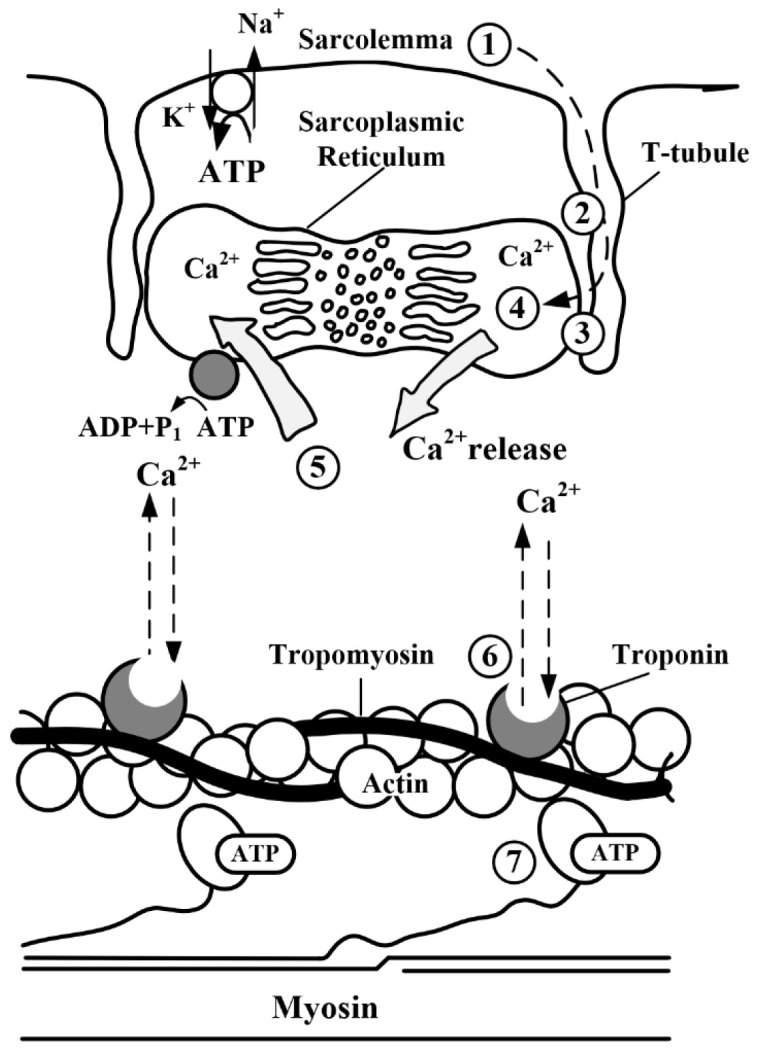
Pathophysiology of muscle fatigue.

Muscle fatigue, defined as a neuromuscular state characterized by declining performance capacity to sustain required/expected force output with subsequent recovery to baseline functionality following rest periods, constitutes a fundamental physiological phenomenon [11,12]. Several studies have established that the deterioration of muscular force-generating capability is primarily attributed to coordinated physiological mechanisms activated during fatigue progression [13,14,15]. As schematized in Figure 1, the classical interpretation maintains that intracellular accumulation of lactic acid and hydrogen ions induces functional impairment of contractile proteins by altering the steric configuration between actin and myosin filaments, resulting in cascading effects, including impaired Ca^2+^ release from the sarcoplasmic reticulum [16,17]. This Ca^2+^ dysregulation is visually represented in the upper quadrant of Figure 1 [18], where compromised ion transport through T-tubules disrupts the excitation–contraction coupling process. These biochemical alterations consequently manifest as detectable variations in sEMG signal characteristics [19].

Muscle fatigue can be biomechanically categorized into static isometric contraction fatigue and dynamic cyclic contraction fatigue based on motor patterns [6]. For fatigue assessment under static isometric conditions, current methodologies predominantly focus on analyzing time-domain features (e.g., integrated electromyography (iEMG), root mean square (RMS)) and frequency-domain features (e.g., mean power frequency (MPF), median frequency (MF)) characteristics of sEMG signals, coupled with advanced classification algorithms to detect fatigue onset [20,21]. During sustained isometric contractions, sEMG signals exhibit distinctive spectral modifications: amplitude augmentation in low-frequency bands (20–60 Hz) accompanied by relative attenuation in high-frequency components (100–500 Hz), with frequency-domain indices demonstrating consistent monotonicity [19]. Specifically, MPF and MF display linear decreasing trends over contraction duration, where the descending rate shows a strong correlation with the fatigue severity level [22,23]. In contrast, time-domain parameters, including iEMG and RMS exhibit proportional linear increases corresponding to neuromuscular activation compensation mechanisms [24].

During dynamic cyclic movements and contractions exceeding 50% of the maximum voluntary contraction (MVC) level, the variability in amplitude and frequency characteristics increases with progressive muscle fatigue [25], thereby diminishing the physical significance of the entire spectrum [1]. Recent investigations have proposed advanced time–frequency analysis methodologies for characterizing muscle fatigue, including wavelet transforms [26,27,28], empirical mode decomposition [29], Gaussian mixture classification [30], and Hilbert transform-based techniques [31,32], which enable effective extraction of sEMG fatigue signatures during dynamic contractions. Additionally, studies have employed machine learning frameworks such as hidden Markov models (HMMs), genetic algorithms (GA), support vector machines (SVM), k-nearest neighbors (KNN), logistic regression (LR), and random forests (RF) for fatigue state classification through EMG signal pattern recognition [29,33]. Notably, the advent of deep learning has facilitated novel applications in physiological information processing [34,35], human intent recognition [36,37], and muscle fatigue classification [38,39], demonstrating enhanced performance in handling complex bio-signal patterns.

To the best of our knowledge, current research has been predominantly focused on fatigue characterization through sEMG signals analysis and fatigue/non-fatigue state classification. At the same time, limited investigations have addressed quantitative characterization methodologies for progressive muscle fatigue states. The establishment of such quantitative frameworks could not only enhance the precision of fatigue state description under constant-force conditions but also improve the estimation accuracy of continuous joint motion parameters, thereby substantially reinforcing the stability of sEMG-based human–robot interaction systems.

This paper proposes a quantitative characterization method for muscle fatigue during cyclical movements based on the high-frequency components of EEMD. The following outlines this paper’s structure: Firstly, the high-frequency components of the sEMG signals of the estimated individual are extracted using ensemble empirical mode decomposition, with the short-time Fourier transform being utilized to determine these components’ median frequency. Secondly, the maximum standard range for muscle fatigue is proposed through linear fitting of the obtained median frequency. Finally, a muscle fatigue estimator is developed to quantify muscle fatigue and complete the experimental verification process, leveraging the standardized maximum range of muscle fatigue.

## 2. Experimental Setup and Signal Preprocessing

The upper limbs of five healthy male subjects were subjected to cyclical movements with terminal loads of 1.25 kg, 3 kg, and 4.5 kg. Each load condition was tested through five parallel experimental trials per participant, with a 1-day interval between trials to eliminate potential training effects on muscle adaptation. This study was conducted in accordance with the Declaration of Helsinki and approved by the academic ethics committee of Hebei University of Technology (Approval number HEBUThmEC2022019). The corresponding physiological parameters of the participants are detailed in Table 1.

**Figure 2:**
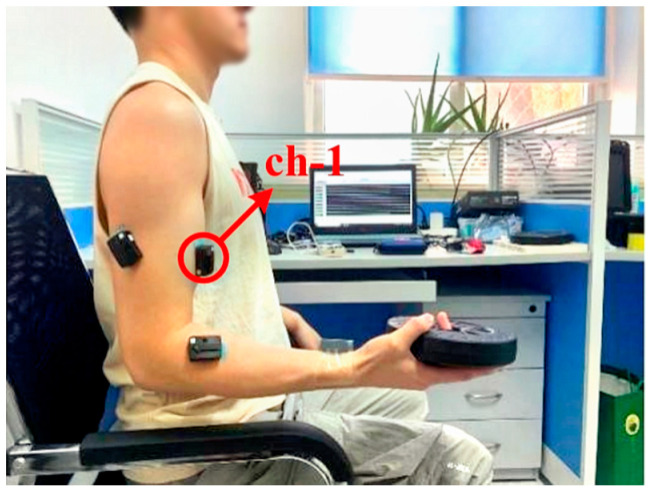
Experimental scenario (ch-1 represents the sEMG signals of the biceps brachii muscle).

The experimental setup is depicted in Figure 2. Since the biceps brachii acted as the dominant activated muscle during this movement protocol, sEMG signals acquisition and fatigue analysis were specifically targeted at this muscle.

The specific experimental process is as follows:

Step 1: The sEMG signals of the biceps brachii muscle related to upper limb elbow joint movement were collected (as required for subsequent research, sEMG signals were collected from four channels in this Figure; this article only focuses on the ch-1 signals). A Delsys dry wireless sensor (Natick, MA, USA) was used, with a sensitivity of 15.6–20.8 mV for the AC input range and 83–1000 mV for the DC input range, and a baseline noise of 0.668–1.125 μV_rms_, with a collection frequency set to 2000 Hz. The sensor also features inter-sensor delay and intra-channel delay both less than 1 sample period, ensuring high data synchronization accuracy. The matching patch locations were cleaned with alcohol before collection.

Step 2: Movements for elbow flexion and extension were performed while the subject’s upper arm remained perpendicular to the ground and their forearm began and ended parallel to the ground. Testers attempted to maintain a duration of 4 s every cycle by using metronome proposals. The end-point loads on the hand were 1.25 kg, 3 kg, and 4.5 kg, respectively. For each group of experiments, 40 cycles were completed if possible. In cases where this was not attainable, the test was carried out to the maximum extent until the muscles were completely fatigued.

Step 3: To prevent training effects from compromising the reliability of the experimental results, each subject was restricted to three experimental sessions per week, with a minimum one-day interval between consecutive sessions. For each participant, five sets of experiments were conducted under each load condition. Randomly, three sets of experimental data were chosen as the training set data, while the remaining two sets were designated as the validation set data.

In addition, to minimize the noise interference in the sEMG signals, the collected signal data were first subjected to band-pass filtering in the range of 10–400 Hz and notch filtering at 50 Hz.

## 3. sEMG Decomposition by EEMD

sEMG is an extremely weak bioelectric signal exhibiting strong nonlinear characteristics. Previous studies have demonstrated that the high-frequency components of sEMG signals possess superior sensitivity for detecting muscle fatigue compared to raw signals, while EEMD shows better performance both in noise reduction and reducing signal non-stationarity [40]. Therefore, in this paper, the EEMD method is employed to decompose the high-frequency components of sEMG signals.

Empirical Mode Decomposition (EMD) is an adaptive time–frequency localization analysis method proposed by Huang et al. [41], which overcomes the limitations of the Fourier transform. EMD decomposes complex signals into a finite number of intrinsic mode functions (IMFs), with each IMF component containing local characteristic signals of the original signal at different time scales. This method performs signal decomposition based on the intrinsic time-scale characteristics of the data itself, requiring no predefined basis functions. Consequently, EMD theoretically applies to the decomposition of any signal type, demonstrating significant advantages in processing non-stationary and nonlinear data.

However, a significant limitation of EMD lies in its susceptibility to mode mixing—a phenomenon where signal components that should inherently belong to distinct IMFs become erroneously blended within a single IMF component. To address this issue, Huang et al. [42] developed the noise-assisted data analysis method known as EEMD. By adding Gaussian white noise to the original data, EEMD compensates for the scale deficiency of the signal, thereby establishing continuity across different scales and reducing the degree of mode mixing. As the added white noise is uncorrelated across multiple iterations, the ensemble averaging process cancels out the noise components while preserving the intrinsic signal characteristics through statistical stabilization.

**Figure 3:**
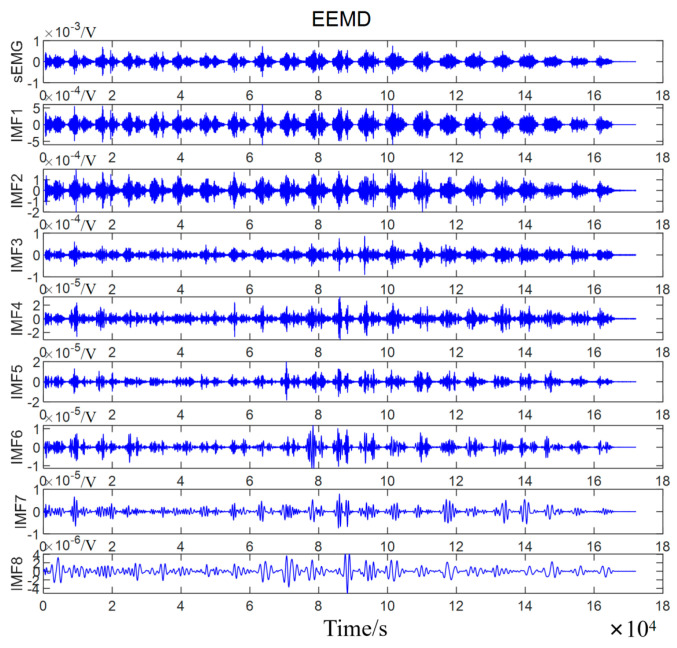
Decomposition characteristics of sEMG via EEMD. The original sEMG signal is decomposed into eight intrinsic mode functions (IMFs) using the EEMD method. The horizontal axis represents time in seconds (s), and the vertical axis represents each IMF component’s amplitude in volts (V). IMF1 corresponds to the highest frequency component, while IMF8 corresponds to the lowest frequency component.

Figure 3 shows the decomposition results of sEMG signals of subject A under a 4.5 kg load by EEMD.

## 4. High-Frequency Component Linear Fitting

The mean frequency (MNF) and MF give fundamental information about the signal spectrum and timing. However, existing studies indicate that MF is less adversely affected by superimposed noise but more significantly influenced by fatigue [43]. Therefore, this study utilized MF to assess regional muscle fatigue in elbow extension exercises. The median frequency is defined as the frequency at which the accumulated spectral energy accounts for half of the total spectral energy, calculated as shown in Equation (1). To capture the complete-cycle spectral features, the decomposed high-frequency components were divided into 10,240 ms time windows with 2560 ms step intervals. The 10,240 ms window size was chosen to ensure sufficient frequency resolution, which is critical for robust median frequency estimation and capturing the progressive spectral shifts indicative of muscle fatigue [44]. The 2560 ms step size strikes a balance between temporal granularity and computational efficiency, allowing for finer tracking of fatigue progression while remaining feasible for analyzing cyclical movements [45]. The power spectral density p(f) within each time window is obtained by using the short-time Fourier transform, and the MF value of each segment is extracted from the power spectral density.

(1) $$\int_{0}^{MF}p(f)df=\int_{MF}^{\infty }p(f)df=\frac{1}{2}\int_{0}^{\infty }p(f)df$$

In the equation, p(f) is the power spectral density of high-frequency components within each time window and MF is the median frequency within this time window.

Further application of the linear regression equation was used to analyze the median frequency distribution throughout the entire exercise period to quantify muscle fatigue performance, The linear regression equation is as follows:

(2) y=Ax+b

where y is the estimated median frequency, A is the slope of the linear regression equation, x is the exercise time, and b is the intercept, corresponding to the initial median frequency.

**Figure 4:**
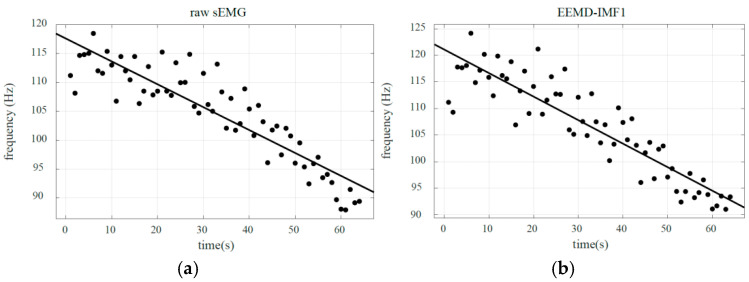
MF distribution of raw sEMG vs. IMF1 at 4.5 kg load: (**a**) raw sEMG; (**b**) IMF1.

The median frequency distribution of the raw sEMG and the median frequency distribution of the high-frequency component IMF1 are shown in Figure 4. We also apply the correlation coefficient (R) to represent the consistency between the median frequency distribution and the fitted line during muscle fatigue. The specific calculation is as follows:

(3) $$R=\frac{\sum_{i=1}^{n}\left(x_{i}-\bar{x})(y_{i}-\bar{y}\right)}{\sqrt{\sum_{i=1}^{n}{\left(x_{i}-\bar{x}\right)}^{2}}\sqrt{\sum_{i=1}^{n}(y_{i}-\bar{y}{)}^{2}}}$$

At the same time, we summarized the linear fitting parameters of all training set data under the 4.5 kg load, as shown in Table 2. It is easy to see that the slope of the high-frequency component IMF1 in EEMD is much less than that of the raw sEMG and other intrinsic modal components. However, research has shown that the lower the slope, the greater the sensitivity to detect muscle weariness [46]. Therefore, this indicates that the high-frequency component IMF1 has a more sensitive ability to detect muscle fatigue. Previous research has shown that the initial and final median frequencies of sEMG under various loads generally remain within a specific range [47,48]. Simultaneously, the table shows that the initial median frequency *mf*_0_ (i.e., *b*) and the final median frequency *mf_S_* at maximum fatigue for each person under each load group are essentially stable within a specific range. To assess the degree of muscle fatigue, we proposed a standardized median frequency distribution range (SMFDR) for sEMG signals during muscle fatigue.

The standardization process was conducted independently for each subject using their training data. This subject-specific calibration accounts for individual physiological differences while maintaining sensitivity to fatigue progression within each participant’s neuromuscular response pattern.

The standardized initial median frequency ${\bar{MF}}_{0}$ under non-fatigued conditions can be calculated by averaging the initial median frequency of the training set data, as described in Equation (4):

(4) $${\bar{MF}}_{0}=\frac{\sum_{i=1}^{n}mf_{0i}}{n}$$

In the equation, ${\bar{MF}}_{0}$ is the standardized initial median frequency, *mf*_0*i*_ is the initial median frequency of the *i*-th set of training data, and *n* is the number of datasets contained in the training set.

The standardized termination median frequency under a complete fatigue state can be calculated by averaging the final median frequencies of the training set data, as described in Equation (5):

(5) $${\bar{MF}}_{s}=\frac{\sum_{i=1}^{n}mf_{si}}{n}$$

In the equation, ${\bar{MF}}_{s}$ is the standardized termination median frequency and *mf_si_* is the termination median frequency of the i-th set of training data. As a result, we define the median frequency distribution range of sEMG signals during standardized muscle fatigue as [${\bar{MF}}_{0}$, ${\bar{MF}}_{s}$].

## 5. Muscle Fatigue Estimator

**Figure 5:**
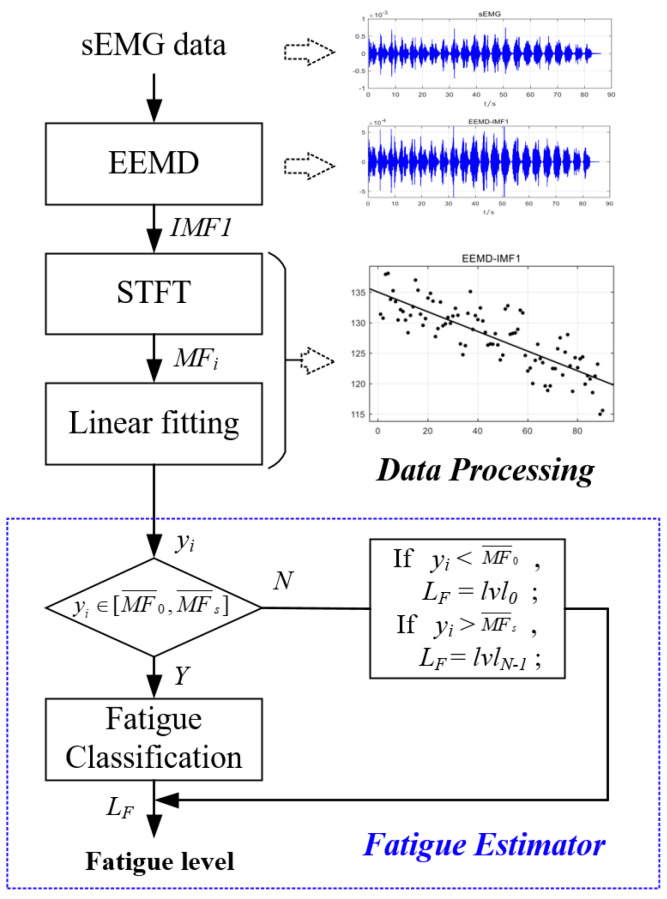
Muscle fatigue estimation process.

**Figure 6:**
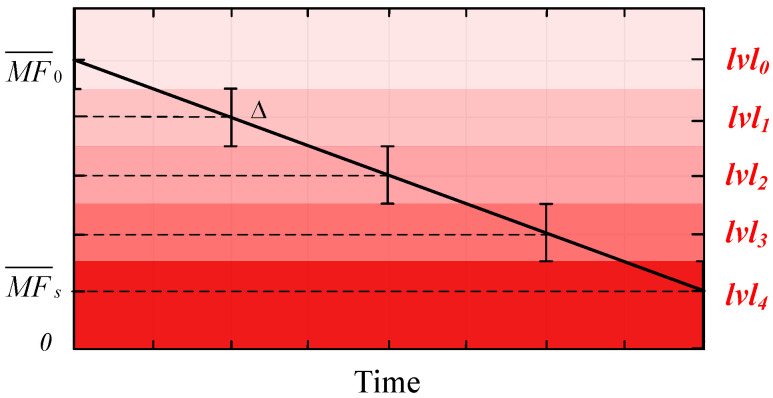
Classification of muscle fatigue level (*N* = 5).

The quantitative characterization framework for muscle fatigue degree proposed in this paper is presented in Figure 5, where the blue region represents the fatigue estimator. The quantification of muscle fatigue degree can be achieved using the normalized MF range derived in Section 4. Specifically, by utilizing the linear regression equation (Equation (2)) established in the preceding analysis, the linearly fitted MF value $y_{i}$ effectively represents the median frequency at the i-th time instance. Then, the median frequency distribution range was divided into *N* portions corresponding to *N* distinct levels of muscle fatigue, as shown in Figure 6. The SMFDR was uniformly partitioned into N discrete intervals (*N* = 5 selected based on preliminary validation of fatigue progression granularity), each corresponding to a distinct muscle fatigue level with an interval width of Δ. This partitioning aligns with established physiological fatigue grading frameworks. Color intensity gradation was implemented, where lighter shades correspond to lower fatigue levels and darker tones represent more pronounced fatigue states, ensuring an intuitive visual interpretation of dynamic fatigue transitions during cyclical movements.

Due to the inherent nonlinear characteristics of sEMG signals, the MF values computed within specified time windows still exhibit nonlinear distributions, as demonstrated in Figure 4. Consequently, we employ the median frequency $y_{i}$ linearly regressed from high-frequency components for muscle fatigue quantification. This frequency must satisfy the following relationship with the muscle fatigue level ($L_{F}$):

(1) When $y_{i}$ ∈ [${\bar{MF}}_{0}$, ${\bar{MF}}_{s}$],

(6) $$\left\{\begin{array}{c} {\bar{MF}}_{0}-\frac{(2L_{F}+1)\Delta }{2}<y_{i}<{\bar{MF}}_{0}-\frac{(2L_{F}-1)\Delta }{2}\\ \Delta =\frac{{\bar{MF}}_{0}-{\bar{MF}}_{s}}{N-1} \end{array}\right.$$

In the equation, *N* is the number of muscle fatigue levels divided; $L_{F}$ is the fatigue level, ranging from 0 to 1, where 0 indicates no fatigue and 1 indicates complete fatigue.

(2) When $y_{i}$ ∉ [${\bar{MF}}_{0}$, ${\bar{MF}}_{s}$],

(7) $$L_{F}=\left\{\begin{array}{lll} 0, & if & y_{i}<\bar{MF_{0}}\\ N-1, & if & y_{i}>\bar{MF_{s}} \end{array}\right.$$

Thus, we can find the $L_{F}$ that matches the fitted median frequency $y_{i}$ at that moment to achieve a quantitative characterization of muscle fatigue.

## 6. Results

**Figure 7:**
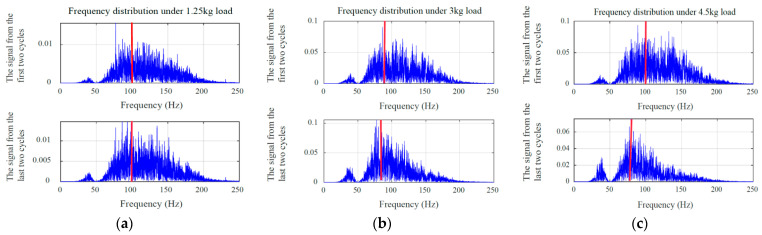
Frequency-domain characteristic variations under different loads: (**a**) 1.25 kg; (**b**) 3 kg; (**c**) 4.5 kg.

Numerous studies have substantiated that during muscle fatigue contractions, the power density in the high-frequency region of sEMG signals decreases, while that in the low-frequency region increases [49,50]. To characterize the progressive changes in muscle fatigue during cyclic movements under varying loads, we performed a comparative analysis of frequency-domain features via Fourier transform analysis. Specifically, spectral analysis was applied to sEMG signals from the first two and last two cycles to detect fatigue-induced frequency shifts, with the results detailed in Figure 7. As can be observed from the figure, the shift from high-frequency to low-frequency components was most pronounced when the load was 4.5 kg, while it was the least noticeable at 1.25 kg. This implies that no significant muscle fatigue occurred during the movement under a 1.25 kg load, whereas the most evident fatigue was observed with the 4.5 kg load. These findings provide an intuitive reference for subsequent experimental validations.

### 6.1. Validation of SMFDR Consistency

**Figure 8:**
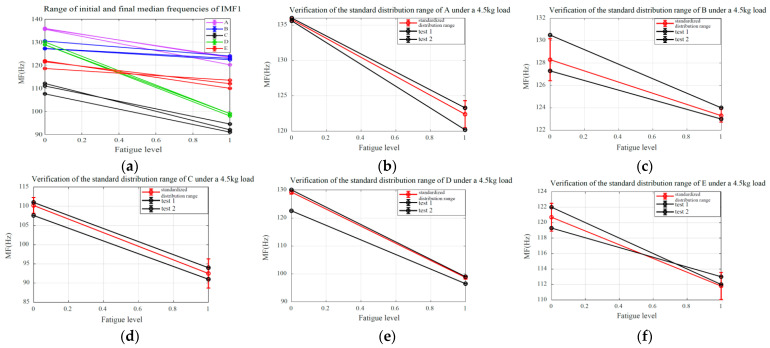
Validation of SMFDR in subjects under 4.5 kg load. (**a**) all subjects; (**b**) subject A; (**c**) subject B; (**d**) subject C; (**e**) subject D; (**f**) subject E.

To validate the consistency and effectiveness of the SMFDR, this study systematically analyzed the initial and final median frequency characteristics of raw sEMG signals and high-frequency component IMF1 for five subjects with 4.5 kg in both non-fatigued and fully fatigued states. Detailed statistical results are presented in Table 3. Analysis of the data presented in the table reveals that while there was significant variability in initial and final median frequencies across different subjects, each individual subject maintained a consistent distribution pattern between their own initial and final values, as illustrated in Figure 8a. Moreover, to further validate the robustness of median frequency distribution ranges under different loads, the initial and final median frequencies of the validation dataset were calculated and compared against the standard ranges presented in Table 3. The results indicated that the initial and final median frequencies of each test were stably distributed around the standardized initial and final median frequencies, respectively, as shown in Figure 8.

In addition, to investigate the distribution patterns of initial and final median frequencies under 1.25 kg and 3 kg loads, as well as their relationship with standardized median frequency ranges, the initial and final median frequencies for these two load conditions were calculated and statistically analyzed, as presented in Table 4 and Table 5. Analysis of the results revealed that under the 1.25 kg load, the initial and final median frequencies exhibited minimal variation and were closely aligned with the initial median frequency of the 4.5 kg load. In contrast, the 3 kg load condition displayed minor discrepancies between initial and final values while maintaining close alignment of the initial median frequency with that of the 4.5 kg load.

### 6.2. Fatigue Quantification and Estimation

**Figure 9:**
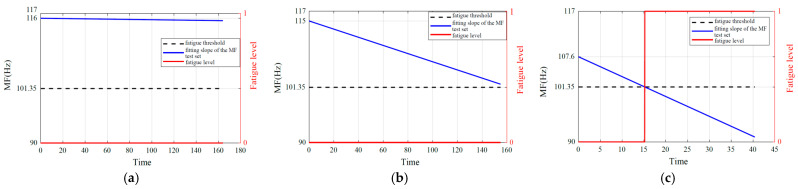
Fatigue estimation results for *N* = 2. (**a**) 1.25 kg load. (**b**) 3 kg load. (**c**) 4.5 kg load.

**Figure 10:**
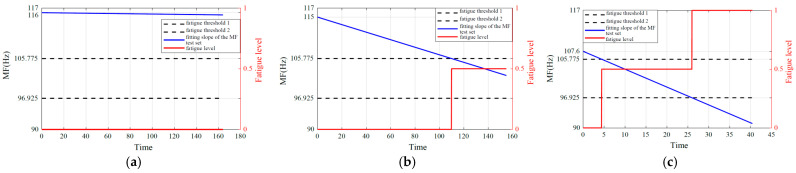
Fatigue estimation results for *N* = 3. (**a**) 1.25 kg load. (**b**) 3 kg load. (**c**) 4.5 kg load.

**Figure 11:**
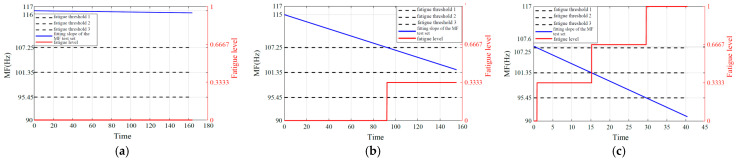
Fatigue estimation results for *N* = 4. (**a**) 1.25 kg load. (**b**) 3 kg load. (**c**) 4.5 kg load.

Based on the analysis and summary of Table 3, Table 4 and Table 5, fatigue estimation is performed on each subject’s validation set data while investigating the impact of fatigue level *N* selection on result reliability. In addition, Figure 7 indicates that muscle fatigue can be perceived through signal frequency shift phenomena. Therefore, the Fourier transform was applied to each dataset prior to muscle fatigue prediction to observe the dynamic fatigue state of muscle changes. Figure 9, Figure 10 and Figure 11 illustrate the fatigue level prediction results under load levels of 1.25 kg, 3 kg, and 4.5 kg when fatigue levels *N* are divided into 2, 3, and 4. The blue line represents the MF fitting line of the high-frequency component of the sEMG signals; the red line indicates the muscle fatigue level progression; the dashed lines demarcate the boundaries between distinct fatigue levels, with *N* = 2 specifically indicating the critical threshold that differentiates fatigue from non-fatigue conditions.

When *N* = 2 (classifying fatigue into two states: non-fatigue and fatigue only):

When *N* = 3 (differentiating non-fatigue, transition, and fatigue states):

When *N* = 4 (differentiating non-fatigue, transition 1, transition 2, and fatigue states):

## 7. Discussion

In view of the impact of muscle fatigue on the accuracy of motion intention recognition during long-term and high-intensity cyclical movements, this paper proposes a method for characterizing the degree of muscle fatigue in cyclical movements based on the high-frequency components of sEMG signals. This method not only improves the detection sensitivity of muscle fatigue but also realizes the quantification of individual muscle fatigue.

To validate the consistency and effectiveness of the SMFDR, this study systematically analyzed the initial and final median frequency characteristics of sEMG signals and their high-frequency component IMF1 in five healthy male subjects under both non-fatigued and fully fatigued states with different loading conditions. The upper limbs of these subjects performed cyclical movements with terminal loads of 1.25 kg, 3 kg, and 4.5 kg, establishing a physiological gradient from light to heavy loading that aligns with typical functional demands while maintaining movement kinematic consistency, as previously documented in the literature [51,52]. Although the current sample included only male participants, existing evidence indicates negligible intergender variations in core muscle fatigue traits [53], which justifies focusing on this demographic for initial validation. Future investigations will expand the participant scope to include female subjects and diverse populations, thereby enhancing the generalizability of these findings.

Due to the significant individual differences in muscle fatigue responses, cross-validation across subjects is not feasible [54]. Each subject’s muscle fatigue response is unique due to factors such as muscle fiber composition, fitness level, and neuromuscular control [55]. These individual differences introduce substantial heterogeneity, making it inappropriate to cross-validate across subjects as it would conflate inter-individual variability with the model’s generalization error. Each subject underwent five sets of experiments per load condition. We selected three sets as training data and used the remaining two sets for validation. This approach was taken to ensure that the validation data provided an independent assessment of the estimator’s performance while minimizing the potential influence of training effects.

Analysis results indicate that the MF of the high-frequency component IMF1 obtained via EEMD demonstrates higher sensitivity and stability in detecting muscle fatigue compared to the raw sEMG signals or the low-frequency components. This finding is consistent with the conclusions drawn by Liu [46]. Improving the sensitivity of muscle fatigue detection can significantly enhance the efficiency of practical applications within the entire muscle fatigue grading estimation process. Based on the analysis of the figures and tables in Section 6.1, it can be concluded that, within a defined time period, the initial median frequency of the same subject under non-fatigued conditions and the final median frequency under fully fatigued conditions each stabilize at distinct yet consistent values. Collectively, these values establish a stable median frequency variation range, which is precisely the standardized median frequency range defined in this study. Based on the analysis of the signal frequency characteristics in Figure 9, Figure 10 and Figure 11 and the fitted slopes, the following conclusions can be drawn: when the load is 1.25 kg, the slope exhibits a slight negative trend with minimal amplitude; as the load increases to 3 kg, the negative shift in the slope becomes significantly more pronounced; when the load reaches 4.5 kg, the slope demonstrates the most substantial negative shift. Further analysis indicates that the quantitative features of the frequency shift phenomenon are highly consistent with the fatigue damage evolution law proposed in this study.

In summary, under identical loading conditions, the standardized median frequency distributions remain stable. Although significant differences exist in median frequency ranges across different loads, their overall distribution trends and coverage intervals remain highly consistent. Notably, the distribution intervals between strictly defined non-fatigued and fully fatigued states demonstrate a marked convergence tendency. Although substantial discrepancies occur between original initial frequencies and standardized initial frequencies under specific loads (1.25 kg, 3 kg, and 4.5 kg), selecting smaller fatigue grade divisions (e.g., *N* = 2 or 3) can effectively reduce fatigue estimation errors. Specifically, when *N* = 3, the estimation accuracy is significantly higher compared to *N* = 2. Considering these factors, the fatigue grading parameter *N* = 3 (comprising non-fatigued, transitional, and fully fatigued states) is recommended as the optimal choice, offering better estimation accuracy and stability.

## 8. Conclusions

This study focuses on the impact of muscle fatigue during prolonged high-intensity cyclic motions on human–robot interaction control, proposing a muscle fatigue characterization method based on the high-frequency components of sEMG signals. This approach improves both the detection sensitivity of muscle fatigue and enables quantitative characterization of individual muscle fatigue states.

(1) Under identical load conditions, the proposed method exhibits a stable and consistent standardized median frequency distribution range. While variations exist in median frequency ranges under different loads, the overall trends and range differences remain relatively consistent and comparable.
(2) The fatigue estimation methodology presented in this study demonstrates superiority in identifying multiple fatigue level states during periodic motion under consistent load conditions. Furthermore, it proves effective in distinguishing three distinct states (non-fatigue, transition, and fatigue) across varying loads.
(3) The quantitative characterization method of muscle fatigue introduced in this work establishes a foundation for integrating muscle fatigue assessment into the continuous motion analysis of human joints in future studies. This approach provides an effective strategy to compensate for the effects of muscle fatigue during human–robot interaction scenarios.

## Figures and Tables

**Table 1:** Physiological information of the subjects.

	Age (year)	Height (cm)	Weight (kg)
A	24	170	55
B	26	175	65
C	24	180	60
D	23	180	56
E	25	173	57

**Table 2:** Linear fitting of the training set for subject *A*.

	*A* (Hz/s)	*mf*_0_ (Hz)	*mf_S_* (Hz)	*R*
sEMG	−0.111 (0.0137)	132.2 (0.231)	121.7 (1.537)	0.777 (0.065)
IMF1	**−0.141** **(0.0184)**	135.8 (0.200)	122.4 (1.916)	0.760 (0.077)
IMF2	−0.077 (0.0147)	84.3 (0.753)	76.9 (0.666)	0.608 (0.088)
IMF3	−0.078 (0.0198)	47.8 (0.811)	40.4 (1.100)	0.564 (0.061)
IMF4	−0.031 (0.0118)	24.5 (0.622)	21.6 (0.611)	0.356 (0.124)

**Table 3:** Statistics of the ${\bar{MF}}_{0}$ and ${\bar{MF}}_{s}$ under 4.5 kg load.

Subjects	${\bar{MF}}_{0}$		${\bar{MF}}_{s}$	
	Raw	IMF1	Raw	IMF1
A	132.2 (0.231)	135.8 (0.200)	121.7 (1.537)	122.4 (1.916)
B	124.1 (1.270)	128.3 (1.877)	120.8 (0.289)	123.3 (0.577)
C	108.5 (2.566)	110.2 (2.348)	89.7 (0.577)	92.5 (1.803)
D	127 (0.520)	129.3 (0.6928)	102 (7.000)	98.7 (0.5774)
E	117.6 (1.365)	120.7 (1.801)	109.3 (1.528)	111.8 (1.756)

**Table 4:** Statistics of the ${\bar{MF}}_{0}$ and ${\bar{MF}}_{s}$ under 1.25 kg load.

Subjects	${\bar{MF}}_{0}$		${\bar{MF}}_{s}$	
	Raw	IMF1	Raw	IMF1
A	127.3 (0.850)	129.9 (1.102)	126.9 (0.520)	129.2 (0.173)
B	127.4 (0.850)	130.5 (0.625)	127.7 (0.265)	130.6 (0.321)
C	118 (2.261)	120 (3.639)	118 (2.454)	119.5 (3.897)
D	125.2 (1.137)	128.2 (1.179)	125.5 (0.755)	128.2 (0.608)
E	123.3 (0.458)	125.5 (0.839)	124.1 (0.723)	126.4 (1.365)

**Table 5:** Statistics of the ${\bar{MF}}_{0}$ and ${\bar{MF}}_{s}$ under 3 kg load.

Subjects	${\bar{MF}}_{0}$		${\bar{MF}}_{s}$	
	Raw	IMF1	Raw	IMF1
A	125.4 (0.907)	128.5 (0.625)	125.2 (0.709)	127.1 (0.902)
B	117.7 (2.818)	120.6 (3.219)	118.8 (5.052)	120.6 (6.577)
C	115.2 (2.558)	115.9 (2.060)	101.8 (3.928)	102.3 (3.799)
D	127.6 (1.069)	130.6 (1.106)	128.1 (0.400)	130.9 (0.208)
E	115.5 (2.250)	117.3 (3.119)	118.4 (2.138)	121.4 (2.601)

## Data Availability

The data and code of the current study can be obtained from the corresponding author upon reasonable request.
